# Epipelagic microbiome of the Small Aral Sea: Metagenomic structure and ecological diversity

**DOI:** 10.1002/mbo3.1142

**Published:** 2020-12-11

**Authors:** Madina Alexyuk, Andrey Bogoyavlenskiy, Pavel Alexyuk, Yergali Moldakhanov, Vladimir Berezin, Ilya Digel

**Affiliations:** ^1^ Research and Production Center for Microbiology and Virology Almaty Kazakhstan; ^2^ Institute for Bioengineering Aachen University of Applied Sciences Jülich Germany

**Keywords:** ecological structure, metagenomics, microbial diversity, shotgun sequencing, Small Aral Sea

## Abstract

Microbial diversity studies regarding the aquatic communities that experienced or are experiencing environmental problems are essential for the comprehension of the remediation dynamics. In this pilot study, we present data on the phylogenetic and ecological structure of microorganisms from epipelagic water samples collected in the Small Aral Sea (SAS). The raw data were generated by massive parallel sequencing using the shotgun approach. As expected, most of the identified DNA sequences belonged to *Terrabacteria* and *Actinobacteria* (40% and 37% of the total reads, respectively). The occurrence of *Deinococcus*‐*Thermus*, *Armatimonadetes*, *Chloroflexi* in the epipelagic SAS waters was less anticipated. Surprising was also the detection of sequences, which are characteristic for strict anaerobes—*Ignavibacteria*, hydrogen‐oxidizing bacteria, and archaeal methanogenic species. We suppose that the observed very broad range of phylogenetic and ecological features displayed by the SAS reads demonstrates a more intensive mixing of water masses originating from diverse ecological niches of the Aral‐Syr Darya River basin than presumed before.

## Graphical abstract

This study aimed to describe and analyze the microbial community from one location of the Small Aral Sea (SAS) using metagenomic approaches. In total 78% of the reads could be assigned to domains of bacteria (2,239,543 reads) and archaea (46,904 reads), while the other 22% of the sequences belonged to eukaryotes and viruses. We presume that the observed broad range of phylogenetic and ecological features displayed by the genetic signatures may demonstrate intensive mixing of water masses originating from different ecological niches of the Aral‐Syr Darya River basin.

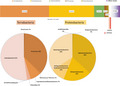

## 1 INTRODUCTION

The Aral Sea, an inland sea in Central Asia, has a multi‐level cyclical history, dating back to about thirty million years, as part of the Paratethys Ocean (Boomer et al., 2000). It was once the world's fourth‐largest lake with an area of 68,000 km^2^. Since the 1960s, the Aral Sea has been shrinking remarkably and recently has been almost depleted by extensive use for irrigation. Nowadays, it represents a highly labile hydrological system consisting of the Small Aral Sea (northernmost part) and the Large Aral Sea (southern part, which is divided into the Eastern Large Aral and the Western Large Aral), with varying degrees of salinity and a maximum of about 40 m of depth in the north part (Izhitskiy et al., 2016; Ltolle et al., 2005). Large spatial and temporal gradients of salinity ranging from 9 g/L to 92 g/L (Izhitskiy et al., 2016) in the Aral Sea create unique and challenging living conditions for all inhabitants, including microorganisms.

In the last several years, there have been many studies documenting the level of salinity, temperature fluctuations, and other physicochemical properties of various parts of the Aral Sea basin (Gaybullaev et al., 2012; Izhitskiy et al., 2014; Rafikov & Gulnora, 2014). Izhitskiy et al. reported that the Aral Sea water bodies, depending on their location and the season, can exhibit very different vertical structures, ranging from a fully mixed to a strongly stratified one. The authors also underlined the dramatic differences in the physical and biological regimes among the different residual basins.

Microbial diversity/structure is a key factor in ecological resilience. Analysis of the Aral Sea microbial communities, an understanding of their features, and their distribution (in the context of environmental conditions) is of paramount importance for environmental monitoring and long‐term remediation strategies in the region (Izhitskiy et al., 2016; Namsaraev, 2018; Shurigin et al., 2019; Stulina et al., 2019). However, a significant part of the lake's microbial community remains unexplored, mainly due to the limited possibilities for the cultivation of microorganisms isolated from hypersaline water.

This study aimed to describe and to analyze the microbial community from one location of the Small Aral Sea (SAS) using metagenomic approaches. This study should complement and extend the data collected in the Large Aral Sea, reported in 2019 by Shurigin et al. (2019). Starting from this pilot run, a group of different SAS locations can be sampled and analyzed in the future. Finally, this study should also contribute to our knowledge concerning the spectrum of microbial metabolic activities of the SAS marine ecosystem. The marine environment remains an immense and mostly untouched source of exclusive microbial metabolites that might be used in novel biotechnological and medical applications (Beygmoradi et al., 2018; Tian & Hua, 2010; Viesser et al., 2020).

## 2 MATERIALS AND METHODS

### 2.1 Water samples collection

**FIGURE 1:**
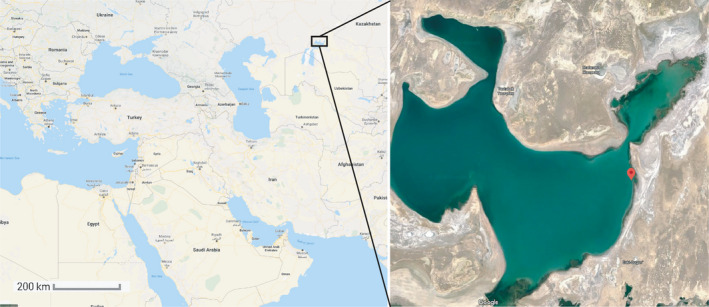
Map of the region where samples were collected in May 2019. The exact site of the sample collection is labeled

Three water samples (20–22°C, salinity 8.1 g/L, approx. 10 L each) were taken from a 1‐m depth of the Small Aral Sea (SAS) in sterile containers at 1‐week intervals during May 2019. The near‐coastal sampling site (46°37′22.0″N 61°28′25.0″E) was chosen as representative, to better capture the epipelagic microbial diversity (Figure 1). This location is considered as ecologically restored and is currently officially used for commercial fishing.

### 2.2 Water samples processing

Immediately after collection, the seawater samples were filtered through a cellulose filter with a diameter of 300 mm and with a pore size of 3 μm to remove zoo‐ and phytoplankton. A similar sample treatment approach has been recently used in the studies by Reddington and by Brumfield who filtered the collected water samples through 1.2 µm and 0.6 μm pore size filters, respectively (Brumfield et al., 2020; Reddington et al., 2020). However, we realize that the removal of suspended solids inevitably depletes the samples of certain microorganisms. The filtrate was then concentrated to a final volume of 500 ml using the tangential flow filtration device Vivaflow 200 (Sartorius Co., Germany) with a 200 cm^2^ polyethersulfone membrane. The samples were pooled and centrifuged at 100,000 *g* for 2 h at 4°C using Avanti J30I ultracentrifuge (Beckman Coulter, USA). The pellet was resuspended in a minimal volume of phosphate‐buffered saline and used for DNA extraction.

### 2.3 Isolation and quantification of nucleic acids

Total DNA was isolated from the sample using the Pure Link genomic DNA extraction kit (ThermoFisher Scientific, USA) and stored at −80°C. Quantitative DNA measurements were performed using the Qubit dsDNA HS (high sensitivity) kit and Qubit 3.0 fluorimeter (both ThermoFisher Scientific, USA), according to the standard manufacturer's instructions. Since only the dsDNA/dsRNA‐bound form of the dye possesses intense fluorescence, there is no interference caused by other species in the solution. The A260/A280 ratio was measured on the Infinite 200 Pro device (Tecan Group Ltd., Switzerland) equipped with the NanoQuant plate (Tecan Group Ltd., Switzerland).

### 2.4 The preparation and purification of genomic libraries

DNA libraries were prepared from 1.0 ng of the obtained dsDNA using the Nextera XT DNA Sample Preparation Kit (Illumina, USA) following the manufacturer's instructions. In brief, the preparation of the libraries included enzymatic fragmentation of DNA, ligation of sequence adapters, preliminary amplification of the library, selection of fractions of the desired length, and clonal amplification of the selected library.

The step “selection of fractions of the required length” was carried out using the Agencourt AMPure XP paramagnetic bead system (Beckman Coulter Corp. USA), capable of binding 100 bp and longer DNA fragments. Excess primers, nucleotides, salts, and enzymes were removed by washing with freshly prepared 80% (v/v) C_2_H_5_OH.

### 2.5 Genomic library quality analysis and bioinformatics

The obtained sequences were analyzed using the 2100 Bioanalyzer system with the DNA 1000 Kit (both Agilent Technologies Inc., USA). For size‐based separation of nucleic acid fragments, they were electrophoretically driven through an interconnected set of specially designed gel‐filled microchannels. The libraries were sequenced using the Illumina MiSeq platform (San Diego, California, USA) using the MiSeq Kit v3, allowing 300‐bp pair‐end readings. The quality of the resulting sequences was tested using the Fast Quality Control (GFastQC) function (https://www.bioinformatics.babraham.ac.uk/projects/fastqc/). Low‐quality reads were excluded, and adapters were trimmed using the Trimmomatic tool (Babraham Bioinformatics, 2019; Bolger et al., 2014). The LCA (lowest common ancestor) algorithm was used for binning short reads onto the nodes of a given taxonomy (such as the NCBI taxonomy), based on alignments.

The presumptive functions and metabolic clustering of the epipelagic microbiota of SAS were analyzed using the KEGG (Kyoto Encyclopedia of Genes and Genomes) orthology database and the database of Clusters of Orthologous Groups of proteins (COGs).

### 2.6 Species taxonomy and diversity analysis

Further bioinformatic processing of the obtained metagenomic data was performed using the Geneious Prime 2019 software (https://www.geneious.com/prime‐features/) and the Kaiju program (http://kaiju.binf.ku.dk/), intended for precise taxonomic classification of readings from high‐throughput metagenomic and metatranscriptomic sequencing. Each read was assigned to a node in the NCBI taxonomy and was labeled by a taxon and the number of matching reads. Due to its protein level classification, the Kaiju algorithm usually achieves higher sensitivity compared to the nucleotide‐based methods (Breitwieser et al., 2019; Kearse et al., 2012; Menzel et al., 2016).

The taxonomy used in this study is mainly based on the List of Prokaryotic Names with Standing in Nomenclature (LPSN; http://www.bacterio.net, accessed on 23.08.2020.) that lists up‐to‐date the validly published names of prokaryotes, under the Rules of International Code of Nomenclature of Bacteria (Parte et al., 2020). For better readability and compactness, some traditional names for groups of phyla (Bacteroidetes (alias FCB Group), PVC, Asgard, DPANN, TACK) are also used in the text, after being properly defined.

## 3 RESULTS AND DISCUSSION

### 3.1 General characterization and phylogenetic diversity of the sequence reads obtained from the SAS

**FIGURE 2:**
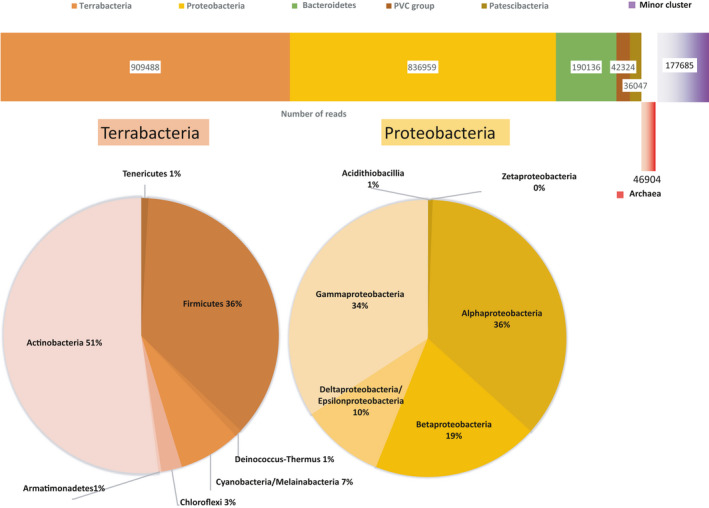
(a) The most common phylogenetic groups of the epipelagic SAS microbiome. The numbers indicate the total number of reads classified to the given group. (b) Relative fractions of the phyla belonging to the group *Terrabacteria*. (c) Relative fractions of the phyla belonging to the group *Proteobacteria*

The extracted DNA (22 ng/µl, A260/A280 ratio 1.8) was subjected to massive parallel sequencing resulting in a database containing 3,248,602 forward sequence reads sized from 35 to 301 nucleotides (overall GC content 46%). After the elimination of low‐quality reads, the remaining 3,206,361 reads ranging from 50 to 286 nucleotides were used for further analysis. In total 78% of the reads could be assigned to domains of bacteria (2,239,543 reads) and archaea (46,904 reads), while the other 22% of the sequences belonged to eukaryotes and viruses. Analysis of the reads by prevalence resulted in their separation into two clusters (major and minor) (Figure 2a). The major cluster comprised 89.97% of the total sequences and included five groups: *Terrabacteria*, *Proteobacteria*, *Sphingobacteria* (*Bacteroidetes* alias FCB group, named after *Flavobacterium*, *Cytophaga*, and *Bacteroides*), *Planctobacteria* (alias PVC group, named after *Planctomycetes*, *Verrucomicrobia*, and *Chlamydiae*), and Superphylum "Candidatus (Ca.) *Patescibacteria*. The minor bacterial cluster (7.93% of total reads) included, besides a homogenous phylum Spirochaetes, a phenotypically very diverse group *Acidobacteria* (whose representatives are mostly uncultivated) and several other phyla. The remaining 2.09% of the identified sequences belonged to archaea.

### 3.2 Phylogenetic and ecological features of the major bacterial groups of the SAS

The reads assigned to *Terrabacteria* and *Proteobacteria* together comprised 84.49% of the identified major cluster sequences. *Terrabacteria* is presumably the most ancient bacterial group of the SAS and includes both Gram‐positive and Gram‐negative strains. Most of the identified sequences within the group belonged to the taxa *Actinobacteria*, *Cyanobacteria*, *Thermi* (*Deinococcus*‐*Thermus*), *Chloroflexi*, *Tenericutes*, and *Firmicutes* (Figure 2b). These phyla share similarities in cellular membrane composition and have many common features in the oxidation/electron transport pathways. *Actinobacteria*, which dominated the SAS *Terrabacteria*, are well known for their ability to decompose “problematic” nutritional substrates, such as chitin, chitosan, and cellulose, and thus play an important role in the trophic chains of the SAS (Souza et al., 2011).

The second‐largest bacterial phylum of the Small Aral Sea *Proteobacteria* was represented in the samples by six taxa, among them a large family of *Enterobacteriaceae* (belongs to *Gammaproteobacteria*) including many marine rhizospheric microorganisms (Figure 2c). This ecological group of microorganisms dominated by *Proteobacteria* inhabits marine sediments and forms distinct communities usually consisting of *Enterobacteria*, *Acidobacteria*, *Actinobacteria*, *Nitrospirae*, *Deltaproteobacteria*, and *Chloroflexi* similar to those found in terrestrial environments (Sogin et al., 2019). According to the same authors, members of these taxonomic groups contribute to the core microbiome living in marine rhizospheres and are predictive of the presence of seagrasses.

**FIGURE 3:**
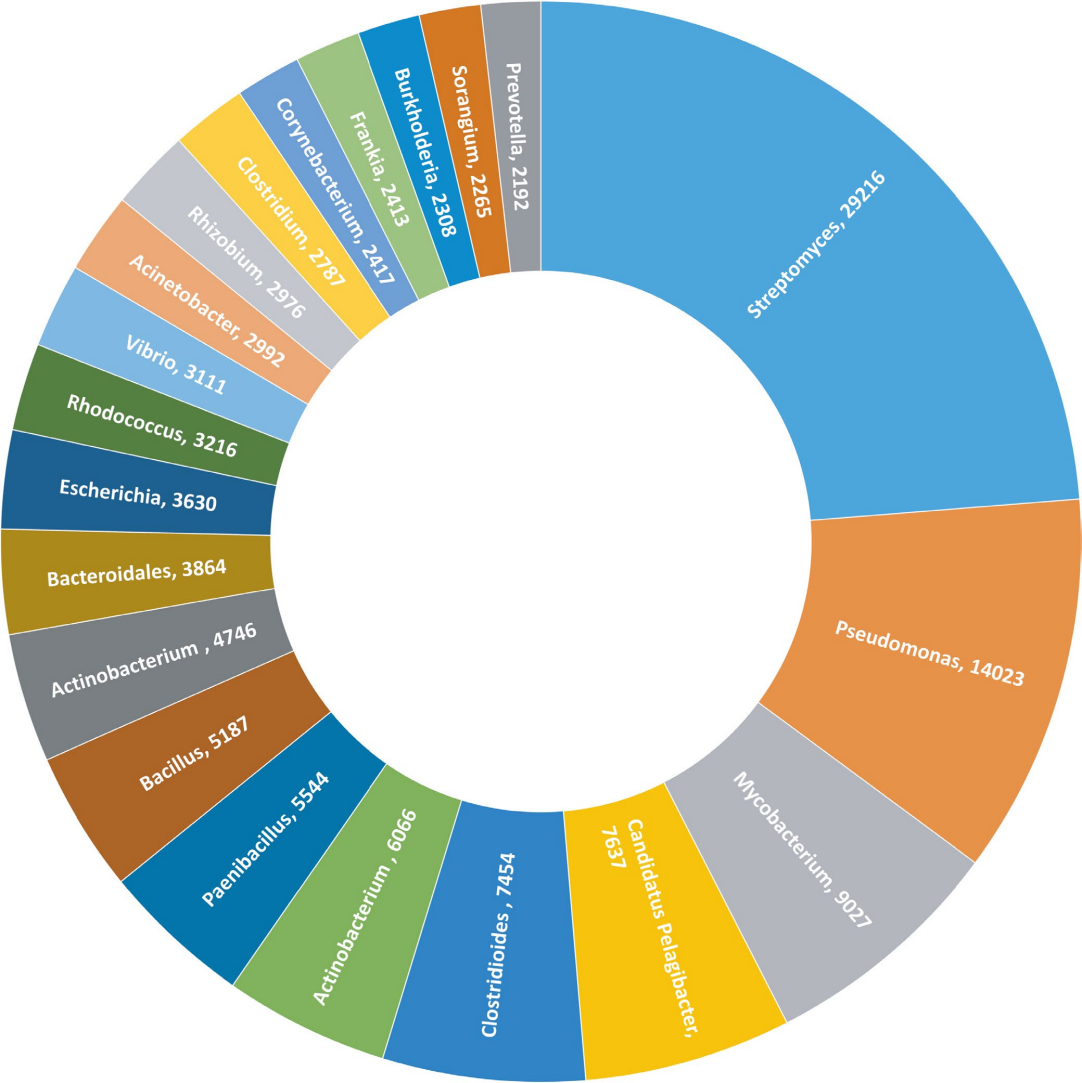
The distribution of most abundant microbial signatures from SAS on the genus level (or the nearest identifiable phylogenetic level) was found using the contig LCA algorithm. The number of hits is displayed after the genus name

Many rhizospheric microorganisms were previously mentioned as typical for the marine plastisphere (Zettler et al., 2013). In this study, we applied the contig‐LCA algorithm on the MG‐RAST server, which finds a single consensus taxonomic entity for all features on each sequence. Indeed, the rhizospheric microorganisms' signatures abundantly appeared on the genus level (Figure 3).

Among the most ecologically interesting *Proteobacteria* found in the epipelagic Small Aral Sea waters were aerobic chemotrophs *Acidithiobacillia* and *Zetaproteobacteria* that use iron and sulfur compounds as their only energy source. Due to their narrow metabolic specialization, these organisms may play a pivotal role in the circulation of Fe/S compounds in the SAS ecosystem.

The FCB group (*Bacteroidetes*) unites heterotrophic Gram‐negative rod‐shaped bacteria capable of gliding locomotion. These bacteria possess multi‐enzyme systems helping to utilize virtually any organic substrates as carbon and energy sources. The members of *Bacteroidetes* display a wide range of other physiological adaptations that allow them to succeed in very diverse aquatic ecosystems (Gupta, 2004). Noteworthy was also the finding in the SAS‐samples sequences specific for the novel class *Ignavibacteria*, belonging to Bacteroidetes. These anaerobic moderately thermophilic bacteria are typically isolated from microbial mats at terrestrial hot springs (Iino et al., 2010). Therefore, their identification in the epipelagic microbiome of the SAS was surprising.

Microorganisms of the PVC group (*Planctobacteria*) which includes *Planctomycetes*, *Chlamydiae*, *Lentisphaerae*, Ca. *Omnitrophica*, Ca. *Poribacteria*, and *Verrucomicrobia* were relatively scarcely represented (1.9%) in the collected water samples. However, *Chlamydiae* may be of great practical interest due to their importance as human and animal pathogens (Sachse et al., 2009).

*Verrucomicrobia* have a low population density but display wide distribution in various freshwater and marine habitats. Some *Verrucomicrobia* possess genes encoding nitrogen fixation and sulfate utilization pathways (Wertz et al., 2012). Their heterotrophic mainly carbohydrate‐decomposing metabolism and the predominantly epibiotic and symbiotic lifestyles imply that these bacteria play a significant ecophysiological and biogeochemical role in the SAS microbiome (Cardman et al., 2014).

Most of *Patescibacteria* representatives, currently grouped into 14 classes, were first discovered by metagenomic analysis of samples from hardly accessible isolated habitats, such as permafrost and deep water trenches (Brown et al., 2015; León‐Zayas et al., 2017; Parks et al., 2018). A particularly high *Patescibacteria* content was later reported for some groundwater microbiomes, reaching 38% of the total reads (Bruno et al., 2017; Schwab et al., 2017). Despite their relatively small number in the epipelagic SAS water (less than 2%) compared to the other “major” groups, they are considered important contributors to the ecological balance of aquatic ecosystems. Ca. *Parcubacteria* and Ca. *Microgenomates* (representatives of superphylum Ca. *Patescibacteria*) seemingly lack common respiratory pathways, which is suggestive of their symbiotic lifestyle (Castelle et al., 2018). However, genetic analysis indicates the involvement of this group in the nitrogen cycle (Danczak et al., 2017; León‐Zayas et al., 2017).

### 3.3 Phylogenetic and ecological characteristics of the minor bacterial and archaeal epipelagic SAS groups

**FIGURE 4:**
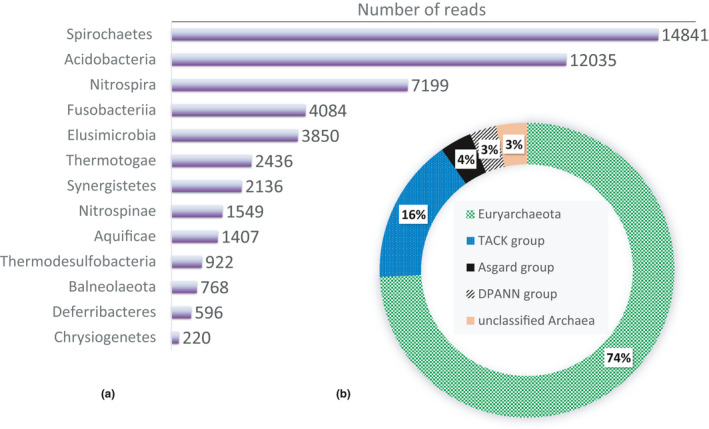
(a) The phyla classified into the minor cluster of bacterial epipelagic SAS microbiome. (b) Relative fraction of different phyla in the archaeal sequences of the SAS

177,568 bacterial reads identified in this study have been reckoned among the “minor cluster” phyla. These 13 phyla include bacteria with very diverse biochemical characteristics, playing very versatile roles in the ecological structure of the Small Aral Sea (Figure 4a).

Remarkable functional specializations and species diversity among representatives of the minor cluster (7.93% of total reads) of the Small Aral Sea sequences is evidence for the rich ecological history of this peculiar drainless salty lake. The distinct phylum *Spirochaetes* is famous primarily for its highly peculiar double‐membrane and helically coiled shape morphology of most of its representatives. These bacteria are also very miscellaneous in their pathogenic capacity and in the ecological niches that they inhabit. The second‐largest group *Acidobacteria* is both phenotypically and physiologically very heterogeneous. The members of this phylum are mostly uncultivated and typically very abundant in soil habitats representing up to 52% of the total bacterial community (Dunbar et al., 2002).

Surprisingly, the minor cluster samples contained numerous signatures of chemolithotrophic microorganisms. Among some most striking findings for us were the sequences typical of hydrogen‐oxidizing bacteria belonging to *Desulfurobacteriales* (*Aquificae)* (Eder & Huber, 2002; Reysenbach & Cady, 2001). Another unanticipated group was *Aquificales*, which representatives prefer rather microaerophilic and thermophilic (>65°C) conditions (Huber et al., 1992).

*Archaea* (Figure 4b) were represented in the samples by all four principal taxonomic groups: (I) *Euryarchaeota*, (II) *Proteoarchaeota* (also known as TACK group named after the initial letters of its early‐found daughter clades *Thaumarchaeota*, Ca. *Aigarchaeota*, *Crenarchaeota* and Ca. *Korarchaeota*), (III) the group comprising Ca. *Lokiarchaeota*, Ca. *Thorarchaeota*, and Ca. *Odinarchaeota*, Ca. *Heimdallarchaeota* (often referred to as Asgard‐group) and DPANN‐group (comprises phyla Ca. *Diapherotrites*, Ca. *Parvarchaeota*, Ca. *Aenigmarchaeota*, Ca. *Nanoarchaeota*, Ca. *Nanohaloarchaeota*, Ca. *Woesearchaeota*, Ca. *Pacearchaeota* and possibly order Ca. *Altiarchaeales*).

The largest known archaeal group, *Euryarchaeota*, accounted for about 74.2% of the analyzed reads. Most of *Euryarchaeota* are extreme halophiles (optimal NaCl concentration 2–4 M), moderate thermophiles (optimal temperature 55°C), and combine several levels of chemolithotrophy. Abundant in the SAS water were strains reducing sulfates to hydrogen sulfide as well as sulfur metabolizing extreme thermophiles—either chemoautotrophs or chemoheterotrophs. Thermoplasma species are obligate thermophiles and acidophiles that are lysed at neutral pH.

The second‐largest group of archaea in the Small Aral Sea was the TACK group (16%), combining chemolithoautotrophs and chemoorganotrophs capable of using elemental sulfur in their metabolism. The number of other archaeal groups altogether did not exceed 10%.

### 3.4 Functional view on the SAS epipelagic microbiome

**FIGURE 5:**
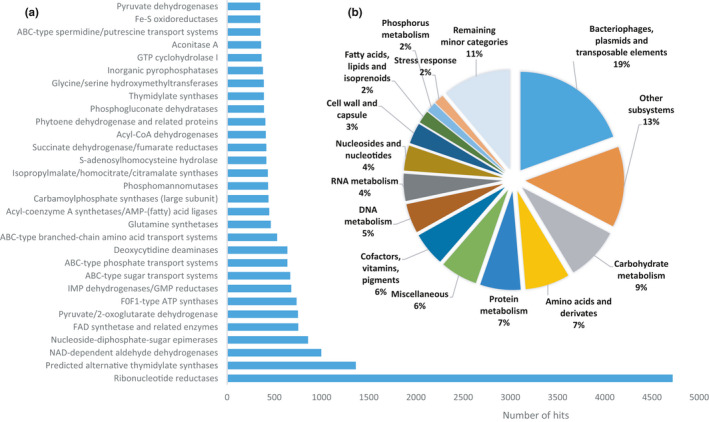
(a) Main predicted protein functions in the SAS microbiota derived from the ortholog analysis (COGs; KEGG). (b) KEGG‐based predictions are colored according to the functional category and sorted in decreasing order of abundance (displayed as numbers)

The functional profile of the microbial community was assessed mainly with the help of the MG‐Rast server using a database of protein orthologous groups (COGs). Each bar in the chart (Figure 5a) indicates the number of reads annotated with predicted protein functions according to the COGs database. The pie chart (Figure 5b) illustrates the distribution of functional categories in the SAS metagenome. According to the obtained results, the largest number of reads belonged to genes encoding functional proteins related to nucleotide pathways. Most prominent among them were ribonucleotide reductases, which are key enzymes mediating the deoxyribonucleotide synthesis, as well as thymidylate synthases, which catalyze the conversion of deoxyuridine monophosphate to deoxythymidine monophosphate.

The SAS samples were reached with functional signatures related to genetic transposable elements (relative abundance 19%), carbohydrate metabolism, as well as to amino acid transport, and protein metabolism. In addition, numerous sequences encoding co‐factors (nucleoside‐diphosphate‐sugar epimerase) were identified.

## 4 CONCLUSIONS

Proper diversity and structure of aquatic microbial communities are of great importance for the sustainability and efficacy of global biogeological transformations. The main object of our pilot metagenomic study was to shed more light on the still poorly discovered epipelagic microbiome of the Small Aral Sea.

The focus was made not only on the prevalence of certain systematic groups but also on their ecological properties and functions. An ecologically remediated near‐coastal location (its well‐being is indicated by the revival of industrial fishing) was chosen as the sampling site. The collected epipelagic samples were tested by massive parallel sequencing without preliminary 16S rRNA amplification, as described elsewhere (Bubnoff, 2008). In our opinion, this high‐throughput method can efficiently provide detailed data on the diversity of the Small Aral Sea microorganisms.

As expected, the majority (44% of the total reads) of the identified DNA sequences of the Small Aral Sea belonged to *Terrabacteria*. This unranked supergroup contains approximately two‐thirds of known prokaryotic species, typically found in aquatic ecosystems. The second‐largest SAS group, *Actinobacteria*, distinguishes itself by being virtually uncultivated and phenotypically very diverse.

The occurrence of some other groups (*Deinococcus*‐*Thermus*, *Armatimonadetes*, *Chloroflexi*) was less anticipated in the epipelagic horizon. Peculiarly, it was found that many detected sequences belonged to strict anaerobes—*Ignavibacteria*, hydrogen‐oxidizing bacteria *Desulfurobacteriales*, and archaeal methanogenic species.

We found that due to the presence of a fairly large number of *Firmicutes*, the taxonomic composition of the sampled SAS‐location displayed a resemblance to the communities in recreational freshwaters of East Fork, Delaware, and Madison lakes in North America rather than that for the Big Aral Sea (BAS) as reported by Shurigin et al. (Lee et al., 2016; Shurigin et al., 2019). In turn, the microbiome of the surface waters of the BAS was more similar to the microbiological communities of Lake Tushchibas (in both deep and shallow parts) (Shurigin et al., 2019). These (dis)similarities possibly reflect the brackish nature of the SAS waters, while the Tushchibas and BAS are rather hypersaline water bodies (Izhitskiy et al., 2016). On the other hand, the sequence analysis of *Archaea* showed about 35% overlapping between BAS and SAS *Halobacteria* species, which points out to the unity of the origin of these microbiomes.

Some specific features exhibited by the SAS microbiome structure included: *Cyanobacteria* were dominated by *Nostocales* and *Oscillatoriophycideae*, while in *Firmicutes*, the amount of *Clostridia* reached about 40%. In comparison with *Actinobacteria* in the lakes of North America being predominantly represented by *Mycobacterium* and *Arthrobacter*, in the SAS microbiome, this group was mainly represented, along with mycobacteria, by *Nocardia*, *Rhodococcus*, *Corynebacterium*, and *Gordonia*. Furthermore, the *Alphaproteobacteria* of the SAS did not encompass *Roseovarius* species, which is common in saline lakes.

We presume that the observed broad range of phylogenetic and ecological features displayed by the genetic signatures may demonstrate intensive mixing of water masses originating from different ecological niches of the Aral‐Syr Darya River basin. On the other hand, the data may reflect the gradual restoration of the ecological balance of the Small Aral Sea after the construction of the Kok‐Aral Dam and a series of dikes built to create spillways to allow the flushing of excess salt (Micklin, 2010).

Future research focus may lay on analyzing the seasonal and yearly dynamics of the bacterial community. As a labile product of a variety of different environmental factors, such as salinity, pH, temperature, osmotic pressure, and solar irradiation, the behavior and the evolution of the SAS ecosystem still needs to be better understood. One of the future tasks will be also to evaluate the exact involvement of different microbial groups in the regional nutrient cycles and biogeochemical flows (both existing and being re‐established). Our present data and future studies should also shed light on the ecological and population dynamics of individual phylogenetic and ecological groups of SAS microorganisms and thus contribute to the economically rational and ecologically sustainable management of natural resources of the whole Aral‐Syr Darya water basin.

## CONFLICT OF INTEREST

None declared.

## AUTHOR CONTRIBUTIONS

**Madina Alexyuk:** Conceptualization (equal); investigation (equal); methodology (equal); validation (equal); writing‐original draft (lead). **Andrey Bogoyavlenskiy:** Conceptualization (equal); funding acquisition (equal); validation (equal); writing‐review & editing (equal). **Pavel Alexyuk:** Data curation (equal); methodology (equal); software (equal). **Yergali Moldakhanov:** Software (equal). **Vladimir Berezin:** Formal analysis (equal); project administration (equal); validation (equal). **Ilya Digel:** Resources (equal); supervision (lead); visualization (equal); writing‐review & editing (equal).

## ETHICS STATEMENT

None required.

## Data Availability

The whole obtained metagenomic data (raw sequence reads) are available on NCBI databases BioProject (PRJNA643916): https://www.ncbi.nlm.nih.gov/bioproject/PRJNA643916, and BioSample (SAMN15435729): https://www.ncbi.nlm.nih.gov/biosample/SAMN15435729, as well as on the SRA database (SRR12265913): https://www.ncbi.nlm.nih.gov/sra/SRR12265913. An interactive HTML file “Figure S1: SAS taxonomic structure,” as well as a raw Kaiju data file “SAS kaiju_taxonpaths,” are available online at https://doi.org/10.5281/zenodo.4057925.
